# Consequences of EEG electrode position error on ultimate beamformer source reconstruction performance

**DOI:** 10.3389/fnins.2014.00042

**Published:** 2014-03-11

**Authors:** Sarang S. Dalal, Stefan Rampp, Florian Willomitzer, Svenja Ettl

**Affiliations:** ^1^Zukunftskolleg and Department of Psychology, University of KonstanzKonstanz, Germany; ^2^Department of Neurology, Epilepsy Center, University Hospital ErlangenErlangen, Germany; ^3^Institute of Optics, Information and Photonics, University of Erlangen-NurembergErlangen, Germany

**Keywords:** electroencephalography, beamformer, head model, inverse problem, source localization, optical 3D metrology

## Abstract

Inaccuracy of EEG electrode coordinates forms an error term in forward model generation and ultimate source reconstruction performance. This error arises from the combination of both intrinsic measurement noise of the digitization apparatus and manual coregistration error when selecting corresponding points on anatomical MRI volumes. A common assumption is that such an error would lead only to displacement of localized sources. Here, we measured electrode positions on a 3D-printed full-scale replica head, using three different techniques: a fringe projection 3D scanner, a novel “Flying Triangulation” 3D sensor, and a traditional electromagnetic digitizer. Using highly accurate fringe projection data as ground truth, the Flying Triangulation sensor had a mean error of 1.5 mm while the electromagnetic digitizer had a mean error of 6.8 mm. Then, again using the fringe projection as ground truth, individual EEG simulations were generated, with source locations across the brain space and a range of sensor noise levels. The simulated datasets were then processed using a beamformer in conjunction with the electrode coordinates registered with the Flying Triangulation and electromagnetic digitizer methods. The beamformer's output SNR was severely degraded with the digitizer-based positions but less severely with the Flying Triangulation coordinates. Therefore, the seemingly innocuous error in electrode registration may result in substantial degradation of beamformer performance, with output SNR penalties up to several decibels. In the case of low-SNR signals such as deeper brain structures or gamma band sources, this implies that sensor coregistration accuracy could make the difference between successful detection of such activity or complete failure to resolve the source.

## 1. Introduction

Any EEG inverse method can only be as good as the forward model input into it (Steinsträter et al., 2010; Acar and Makeig, 2013). Despite the incredible amount of research effort aimed toward reducing source localization error with improved head models and inverse techniques, one significant source of error is quite mundane yet remains prevalent.

With any EEG study requiring precise source localization, EEG electrode positions must somehow be coregistered onto each subject's structural MRI. This is typically done with the placement of fiducial markers at known anatomical locations, which are then localized using a 3D digitization stylus. Some laboratories acquire this data together with a digitized headshape to further improve fit to an MRI-derived headshape.

Each EEG electrode position must additionally be digitized relative to these fiducial points. The corresponding fiducial locations must then be located on the subject's MRI. Most EEG researchers simply estimate the fiducial locations on the image volume by eye on orthogonal MRI slices, a procedure prone to error as well as inter-experimenter variability. A digitized headshape, when available, can help reduce this error, but certain hairstyles or the thickness of the electrode cap can reduce the reliability of this procedure. Additionally, the digitization system itself introduces its own measurement errors (Le et al., 1998).

When these data are to be used for source localization, a common assumption is that the localized sources will be subject to a corresponding displacement error proportional to the sum of the aforementioned errors. This may indeed be the most obvious effect with traditional dipole source localization methods (De Munck et al., 1991; Khosla et al., 1999; Wang and Gotman, 2001; Beltrachini et al., 2011; Acar and Makeig, 2013), especially if the displacement error for each sensor tends to have similar magnitude and direction. However, measurement errors from the digitizer may not necessarily be in a consistent direction for each electrode. Additionally, source reconstruction methods based on spatial filtering, such as beamforming (van Veen et al., 1997), particularly rely on accurate forward models for optimum performance (Hillebrand and Barnes, 2003, 2011). We therefore hypothesized that the end result of sensor coregistration error on beamformer performance would ultimately be an effective error in the forward model with a more complex impact on source reconstruction quality.

**Figure 1:**
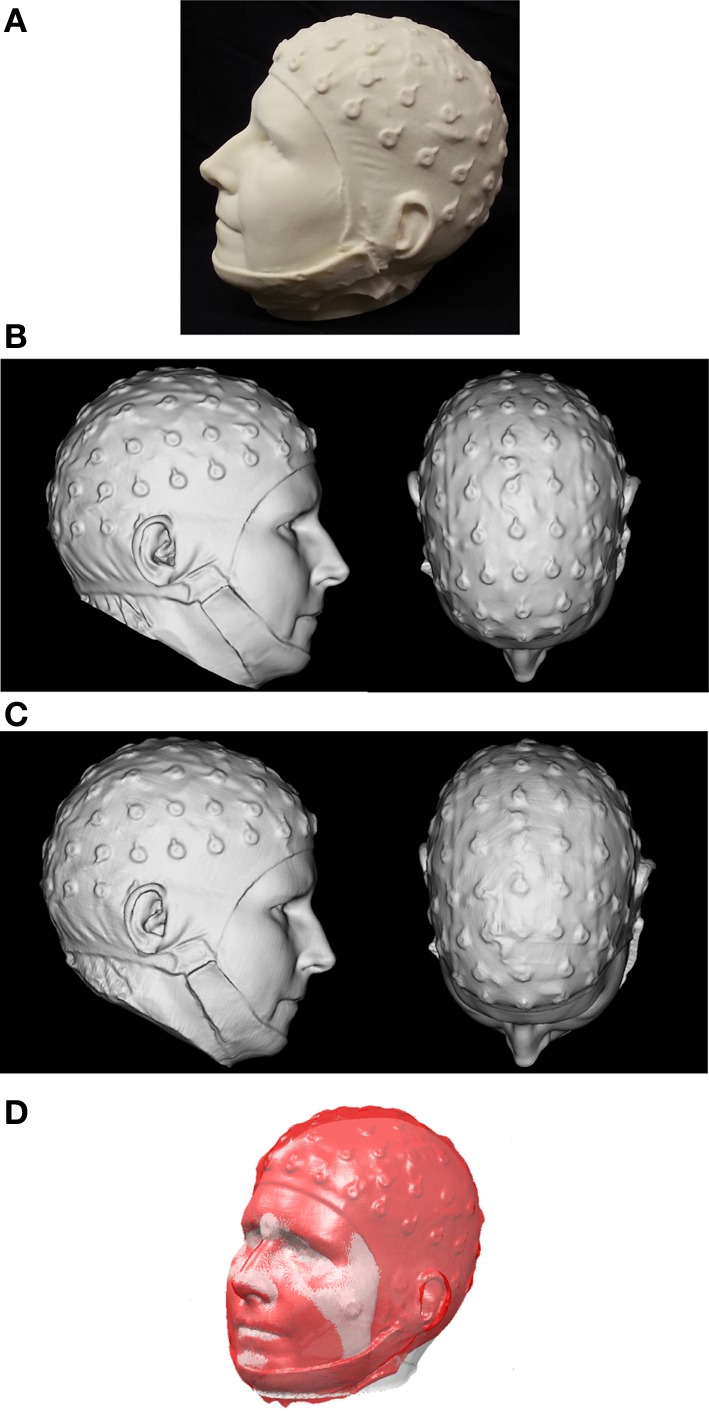
**(A)** The 3D-printed replica head, created from first scan with the FaceSCAN^3D^ sensor of a real human subject wearing an EEG cap **(B)** 3D mesh of the printed model acquired with the FaceSCAN^3D^ sensor from which a “ground truth” coordinate list of electrode positions was generated. **(C)** 3D mesh of the printed model acquired with the FlyTri sensor. **(D)** Registration of EEG electrode positions from the 3D FlyTri model to structural MRI. The gray surface is generated from the segmented MRI and the red surface is generated from the FlyTri scan of the printed model.

In this work, we shall demonstrate the impact that realistic sensor registration inaccuracies have on synthetic EEG data derived from a 3D-printed head model of actual size generated from a high-resolution 3D scan of a real human head. Optical 3D sensing devices have become affordable enough to provide a potential alternative to traditional electromagnetic digitizers (Koessler et al., 2011). With such a technique, EEG electrodes are readily apparent in the resulting 3D surface visualization (see Figure 1). A coordinate transformation based on facial surfaces or landmarks allows these scanned EEG electrode positions to be easily coregistered to the MRI.

We evaluate our method for fast and accurate registration of EEG electrodes to 3D MRI reconstructions using the “Flying Triangulation” (FlyTri) 3D sensor (Ettl et al., 2012, 2013) as well as the commonly used Polhemus Fastrak electromagnetic 3D digitizer. With measurement of the 3D-printed replica head, we restrict our assessment to errors resulting from intrinsic measurement noise and transformation into MRI space. A previous study found a mean electrode localization error of 3.6 mm with the Fastrak digitizer (Le et al., 1998). Our group previously reported a comparable intrinsic measurement error of 3.4 mm (Ettl et al., 2013), while another paper reported an overall mean error (between Polhemus measurements and corresponding MRI markers) of 8.7 mm (Whalen et al., 2008). In this paper, we first measure the coregistration accuracy for both FlyTri sensor and the Fastrak, and then quantify the ultimate impact of their respective coregistration errors on beamformer performance.

## 2. Materials and methods

A 68-electrode EEG cap (Sands Research Inc., El Paso, TX, USA) was placed on an adult human subject. A high-resolution 3D scanner employing fringe projection (FaceSCAN^3D^, 3D-Shape GmbH, Erlangen, Germany) was used to digitize the subject's head with the electrode cap in place; this device has 0.1 mm measurement uncertainty. Its lateral resolution is 1 mm and it has a 600 mm × 400 mm field of view for a 400 mm depth of field. The final 3D digital mesh has a resolution of 0.5 mm by subdivision of the 3D data. The full fringe projection scan formed the basis for a 3D mesh that was used to “print” a true-to-size 3D replica of the subject's head wearing the electrode cap (Figure 1A). The FaceSCAN^3D^ sensor was then employed again to scan the 3D-printed model and generate a dense digital mesh, in order to eliminate the effect of any potential imperfections in the 3D printing process.

Two researchers then manually determined electrode positions in software from the 3D digital mesh, by visually identifying the center of the electrode shapes (Figure 1B). The average of these two coordinate lists was used as the “ground truth” for the purposes of this study.

EEG electrode positions were then measured on the 3D-printed model using the common Fastrak electromagnetic digitizer (Polhemus Inc., Colchester, VT, USA) as well as the OSMIN laboratory's Flying Triangulation (FlyTri) face sensor (Willomitzer et al., 2010). The FlyTri was chosen because of its motion-robust ability to acquire 3D data of complex objects by hand-guiding the sensor around the object while displaying the current measurement progress in real time.

The Fastrak measurements were performed in the center of a magnetically shielded room (designed for MEG acquisition) with no metal objects in the vicinity. The measurement was performed twice to yield two datasets; five electrodes which were either not captured or for which the two measurements differed by more than 10 mm were rejected from further analysis, and the two measurements for each remaining position were averaged for subsequent use. The FlyTri data were meshed (Materialise Inc., Leuven, Belgium) and, as with the fringe projection data, were then visualized as a surface in software (Figure 1C). Two researchers independently identified electrode centers from this visualization manually; these two coordinate lists were also averaged.

The MRI head surface (scalp) mesh was generated using BrainVisa (http://brainvisa.info/). FaceSCAN^3D^-MRI and FlyTri-MRI coordinate transformation matrices were computed as follows. An initial rigid body transformation matrix between FaceSCAN^3D^ or FlyTri coordinates and MRI space were computed through the use of anatomical fiducials as control points on each tessellated head surface: center of each eye, nasion, nose tip, each inferior corner of the nose, the apex of each ear, and the inferiormost point of each ear. These anatomical features were chosen as they are less susceptible to movement and are not (typically) covered by hair. This initial transformation matrix was then refined using the Iterative Closest Point (ICP) algorithm, using the points from each surface that form the upper facial features, i.e., forehead, eyes, nose, and cheeks (Figure 1D) (Koessler et al., 2011). These transformation matrices were then applied to the previously obtained electrode coordinate lists from the FlyTri and FaceSCAN^3D^ data to obtain their corresponding locations in MRI space.

For the Fastrak coordinates, a rigid body transformation was performed based on the manual annotation of the three fiducial locations (the left and right preauricular points and the nasion) on the subject's MRI using the NUTMEG toolbox (Dalal et al., 2011b).

Brain, skull, and scalp surfaces were generated from the subject's MRI using BrainVisa. Realistic EEG head models were then constructed from these tissue surfaces with OpenMEEG (Gramfort et al., 2010, 2011), to generate a three-layer BEM-based head model. A brain:skull:scalp conductivity ratio of 1:0.067:1 was used, as recommended in Oostendorp et al. (2000). Using a 5 mm grid covering the brain space, lead fields were generated for each of the three sets of sensor coordinates: FaceSCAN^3D^, Fastrak, and FlyTri.

Simulated sources were synthesized using the NUTMEG toolbox. In individual simulations, a dipole source consisting of a 19 Hz oscillation (Figure 3A) sampled at 1000 Hz was placed at each location on the grid and projected to the EEG electrode array via the BEM-based forward model constructed with the fringe projection electrode coordinates, which was taken to be the ground truth. (Only the 63 electrodes that were retained after the Fastrak digitization procedure mentioned above were used for all simulations.) Gaussian noise was added to the channels to yield simulated EEG datasets with signal-to-noise ratios ranging from −30 to +30 dB. For each dipole simulation, the sources were then reconstructed over both space and time using an LCMV beamformer (as implemented in NUTMEG) and BEM-based forward models from the Fastrak coordinates and from the FlyTri coordinates. No temporal filtering or matrix regularization was applied prior to beamformer computation.

The output SNR was estimated at every voxel by differencing the rms of the 150 ms window containing the oscillation by the rms of a corresponding 150 ms baseline window, and normalizing by the baseline rms. The voxel with the maximal output SNR was selected, and its distance from the simulated source location was computed. To summarize the overall effects of input SNR on beamformer performance, the peak output SNR was averaged for simulated sources placed at every point in the voxel grid.

## 3. Results

**Figure 2:**
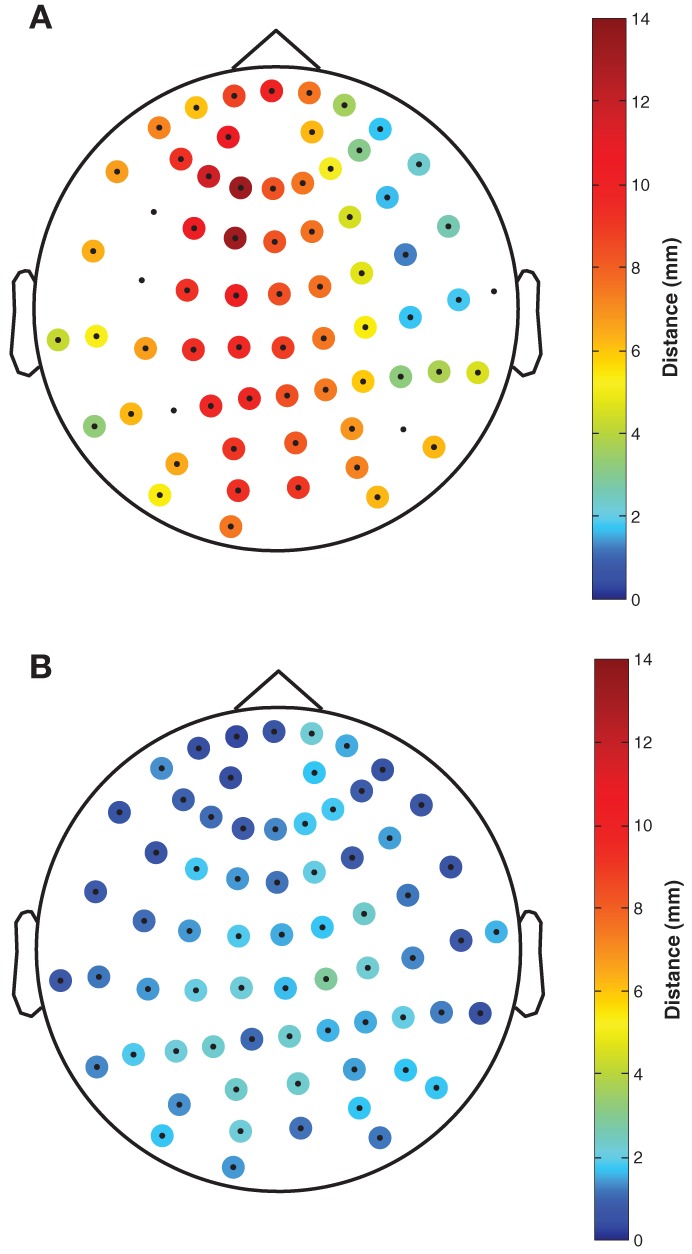
**The distance of (A) Fastrak and (B) FlyTri electrode coordinates, relative to the fringe projection coordinates taken as the gold standard, is indicated by colors at each electrode position**. Coordinates rejected from the Fastrak dataset due to missing data or intermeasurement discrepancies greater than 10 mm are shown with no color. The Fastrak coordinates had a mean deviation of 6.8 mm, while the FlyTri coordinates had a deviation of only 1.5 mm.

The mean deviation of registered Fastrak electrode positions relative to the fringe projection electrode positions was 6.8 mm (range: 1.3–13.3 mm, see Figure 2A). The mean deviation of registered FlyTri electrode positions relative to fringe projection was 1.5 mm (range: 0.5–2.9 mm, see Figure 2B). These deviations comprise both intrinsic measurement errors as well as MRI coregistration errors, and therefore represent realistic errors that might occur in practice with each technique.

**Figure 3:**
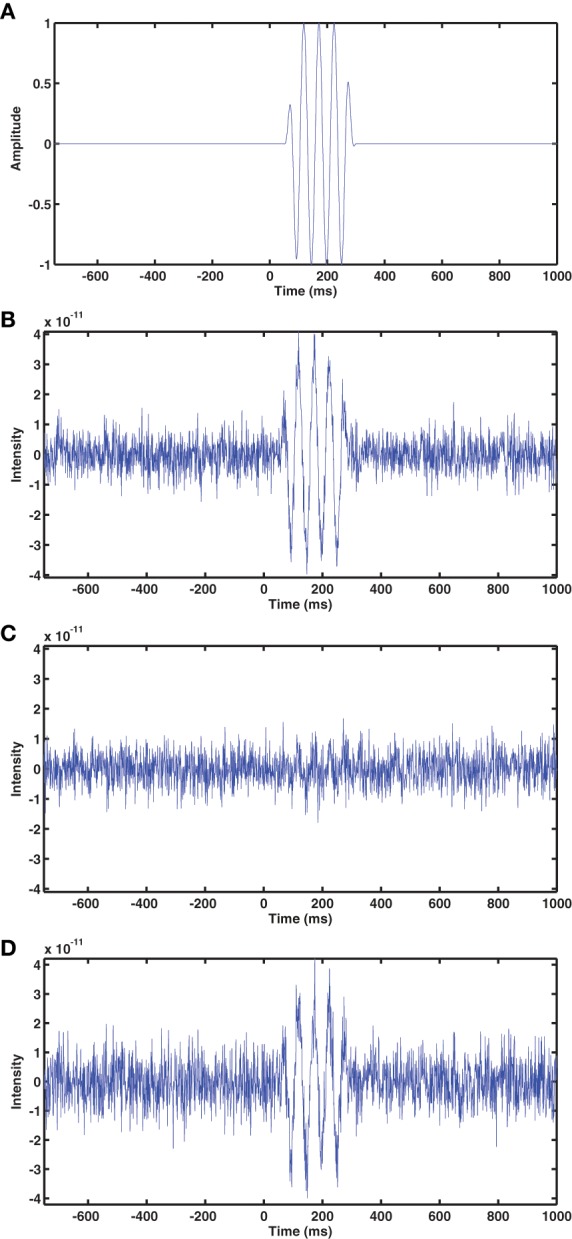
**(A)** The time series of the simulated source, which was placed at a location in the left hippocampus. Gaussian noise was added to the simulated EEG channels such that the SNR was −5 dB in this example. **(B)** The beamformer time series at the source location, determined with the EEG coordinates originally used to generate the simulation (measured via fringe projection). **(C)** The beamformer time series reconstructed using the Fastrak-based electrode coordinates. **(D)** The beamformer time series reconstructed using the FlyTri-based electrode coordinates. The FlyTri-based reconstruction has a visibly higher SNR than the Fastrak-based reconstruction.

**Figure 4:**
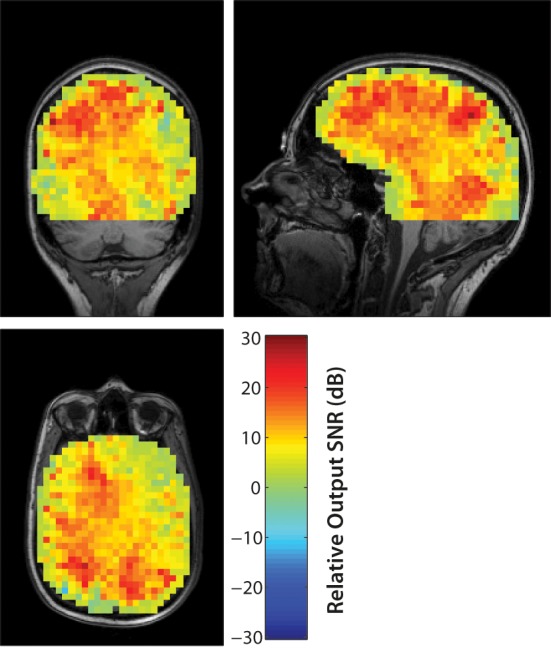
**The improvement in the output SNR of FlyTri-based reconstructions over Fastrak-based reconstructions are mapped here as a function of simulated source location, at an input SNR of −5 dB**. The improvement covers nearly all brain regions, with no clear relationship to source depth.

The output SNR of beamformer reconstructions had a clear relationship to sensor coregistration accuracy. An example of beamformer reconstructions with a source in the left hippocampus is shown in Figure 3. The simulated EEG data were generated with the fringe projection coordinates, so a reconstruction using these same coordinates should be considered the upper bound on beamformer reconstruction quality (Figure 3B). Fastrak-based beamformer results (Figure 3C) exhibited higher noise levels than the FlyTri-based results (Figure 3D). This was the case for nearly all brain regions, with source depth bearing no clear influence on this relationship (Figure 4).

**Figure 5:**
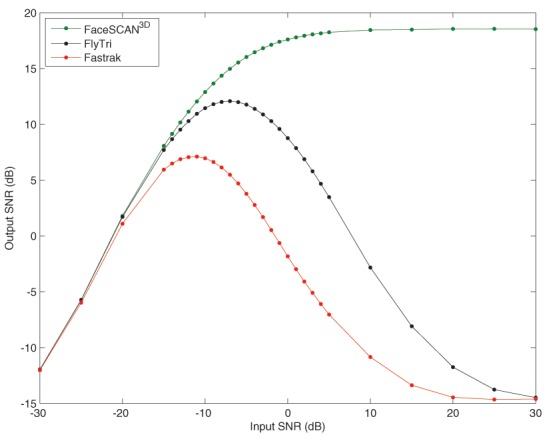
**The mean beamformer output SNR averaged over all simulated source locations as a function of input SNR**. The output SNR from fringe projection (i.e., the same electrode coordinates from which the simulation was generated) increases with input SNR, reaching an asymptotic limit of approximately 18.6 dB. When reconstructing with FlyTri and Fastrak coordinates (i.e., with electrode position measurement errors), output SNR improves to a point but then falls with further increases of input SNR. Nevertheless, the FlyTri coordinates outperform the Fastrak coordinates by several decibels, above an input SNR of approximately −20 dB.

To quantify the results for arbitrary source locations and as a function of input SNR, subsequent analyses were performed on the average across all of the simulated source locations covering the brain grid. The output SNR from fringe projection (i.e., the same electrode coordinates from which the simulation was generated) increases with input SNR, reaching an asymptotic limit of approximately 18.6 dB, averaged across all simulated source locations (Figure 5). Similar to source localization error, when using FlyTri or Fastrak electrode coordinates, the resulting output SNR increases to a point with input SNR, but then counterintuitively falls with further increases of input SNR. Nevertheless, the FlyTri-based source reconstructions outperform the Fastrak-based reconstructions by several decibels, above input SNRs of approximately −20 dB.

With an input SNR of −5 dB, source reconstructions based on the Fastrak-registered electrode coordinates exhibited an average output SNR degradation of −12.2 dB relative to reconstructions based on the original coordinates used to perform the simulation. In contrast, FlyTri-registered electrode coordinates resulted in beamformer output degraded by only −4.3 dB. The difference in output SNR across the full range of input SNRs can be seen in Figure 5.

**Figure 6:**
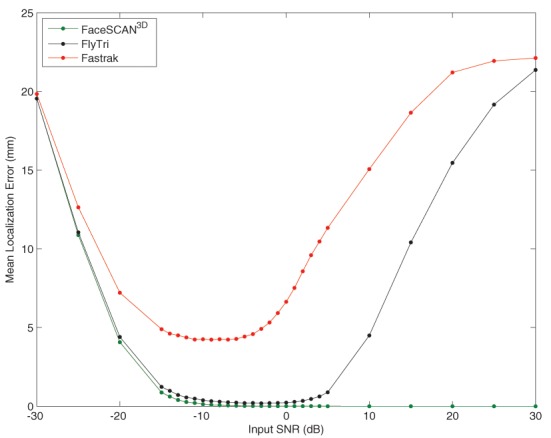
**The ultimate source localization error for each set of electrode coordinates was assessed at each input SNR**. Shown here is the mean localization error across sources simulated at each location of the voxel grid. As with output SNR, the source localization performance with Fastrak and FlyTri coordinates improves but then deteriorates again with further increases of input SNR. However, the source localization with FlyTri coordinates approaches negligible error (<1 mm) for a wide range of input SNRs, in contrast to the Fastrak results.

At optimal input SNRs between −15 and −3 dB, the mean localization error of the Fastrak-based beamformer reconstructions was between 4 and 5 mm, comparable to the sensor registration error. In contrast, fringe projection and FlyTri coordinates approach negligible source localization error (<1 mm) at input SNRs of −15 and −14 dB, respectively. However, again, both Fastrak- and FlyTri-based reconstructions counterintuitively resulted in gradually increasing localization errors at higher input SNRs, though the FlyTri-based results were consistently better and were robust to a wider range of input SNRs (see Figure 6).

## 4. Discussion

The beamformer is known to be sensitive to head model deficiencies (Hillebrand and Barnes, 2003; Steinsträter et al., 2010; Stahlhut et al., 2012). Our results demonstrate that simple sensor coregistration error impacts head model quality enough to have important consequences for beamformer reconstruction performance. The measurement error associated with widely used electromagnetic digitization devices ultimately deteriorates beamformer performance. This not only impacts source localization uncertainty, but, perhaps more importantly, output SNR. In other words, inaccurate sensor coregistration (and, by extension, poor head model quality) does not only result in mislocalization of source locations, but may prevent detection of weaker signals entirely.

In practice, electromagnetic digitizer performance can be impacted by additional sources of error than reproduced here, including head movements and mechanical skin displacement from stylus pressure, so one can expect potentially larger SNR degradation with live human subjects. Optical 3D sensors, on the other hand, require no physical contact with the subject and are usually designed to be robust to any head motion, so performance in practice should not differ significantly from the results presented here.

Therefore, accurate sensor coregistration should improve the detectability of high-frequency activity related to healthy function (Dalal et al., 2008; Fries et al., 2008; Jerbi et al., 2009; Dalal et al., 2011a, 2013b), psychiatric disorders such as schizophrenia (Herrmann and Demiralp, 2005; Uhlhaas and Singer, 2010), and neurological disorders such as epilepsy (Jacobs et al., 2010; Rampp et al., 2010). Although source depth did not directly impact beamformer performance in this study when controlling for input SNR, in practice deep sources also generate weaker signals. This implies that beamformer reconstructions made with the most accurate sensor positions should additionally offer the most “reach” into the brain. Therefore, the detectability of deeper brain structures like the hippocampus (Quraan et al., 2011; Dalal et al., 2013a) should benefit from more accurate sensor coregistration.

These results, by extension, speak to the importance of measuring electrode positions in individual subjects and coregistering them to individual structural MRIs, rather than relying on generic templates. However, the most common electrode localization technique using electromagnetic digitization technology is somewhat time-consuming and still exhibits enough measurement error to detrimentally impact ultimate beamformer performance. In recent years, 3D sensing technology has both developed rapidly and become more affordable, providing a viable alternative that is simultaneously more time-efficient and accurate (Koessler et al., 2011; Ettl et al., 2013).

While outside the scope of the present study, the surface area of EEG electrodes (Wendel et al., 2007) may also be a parameter that is important to incorporate into lead field models used in conjunction with beamformers. Though EEG electrodes are generally assumed to be point detectors, in reality they measure the averaged surface potential over the contact area. This may be a negligible factor for traditional EEG electrode caps (Nelson and Pouget, 2012), but could have significant consequences for active electrodes and dry electrodes, which tend to have larger surface areas than traditional electrodes, as well as for studies involving small children, in which electrodes may cover a larger proportion of the head surface (Ollikainen et al., 2000; Pursiainen et al., 2012).

One implication of the present study is that high input SNR may actually degrade beamformer reconstructions in the presence of realistic sensor coregistration error. This finding is in line with previous studies characterizing the phenomenon of beamformer “mismatch” (Cox, 1973; Godara, 1986), in which inaccuracies of forward models result in signal cancelation at high input SNRs for generic beamformer applications. In applications to EEG/MEG signals, our finding complements (Hillebrand and Barnes, 2003), which found that source orientation error (resulting from either MEG-MRI coregistration error or imperfect MR segmentation error) deteriorates the performance of a cortically-constrained beamformer in high SNR conditions. Essentially, the selectivity of the beamformer spatial filter becomes sharper with higher input SNR, eventually resulting in cancelation of the desired signal when the lead field model deviates from the true lead field (Cox, 1973; Hillebrand and Barnes, 2003). Our finding generalizes the interaction of input SNR and coregistration error to the standard LCMV beamformer (without cortical constraints) in EEG. This phenomenon could explain some cases of source reconstruction failure, and suggests that minimizing coregistration error may also be important when attempting to reconstruct the strongest EEG sources.

In conclusion, the seemingly mundane issue of localizing EEG electrodes has a substantial impact on head model quality and ultimate beamformer source reconstruction performance, penalizing output SNRs by several decibels. Researchers in pursuit of weaker neural signals, in particular, should take measures to minimize these errors as well as use realistic head models in order to optimize beamformer performance and improve the likelihood of detecting these sources. New technologies based on optical 3D sensing, such as the FlyTri sensor employed in this study, have lower measurement noise and reduce error resulting from MRI coregistration since the scanned facial features can be directly matched to MRI-derived facial surfaces, resulting in more accurate coordinate transformation to MRI space. Combining this improved registration performance with realistic EEG head models should yield tangible improvements in output source reconstructions.

### Conflict of interest statement

The authors declare that the research was conducted in the absence of any commercial or financial relationships that could be construed as a potential conflict of interest.
